# GPS tracking methods for spatiotemporal air pollution exposure assessment: comparison and challenges in study implementation

**DOI:** 10.1186/s12942-025-00405-x

**Published:** 2025-07-26

**Authors:** Kalliopi Kyriakou, Benjamin Flückiger, Danielle Vienneau, Nicole Probst-Hensch, Ayoung Jeong, Medea Imboden, Aletta Karsies, Oliver Schmitz, Derek Karssenberg, Roel Vermeulen, Gerard Hoek, Kees de Hoogh

**Affiliations:** 1 Institute for Risk Assessment Sciences (IRAS), Division of Environmental Epidemiology, Utrecht University, Utrecht, the Netherlands; 2 Aristotle University of Thessaloniki, Thessaloniki, Greece; 3 Swiss Tropical and Public Health Institute, Allschwil, Switzerland; 4 University of Basel, Basel, Switzerland; 5 Department of Physical Geography (Geo), Utrecht University, Utrecht, the Netherlands

**Keywords:** GPS tracking, Time-activity diaries, Tracking campaign design, Smartphone app, External GPS device, Air pollution

## Abstract

**Background:**

Epidemiological studies investigating long-term health effects of air pollution typically only consider the residential locations of the participants, thereby ignoring the space-time activity patterns that likely influence total exposure. This paper, part of a study in which residential-only and mobility-integrated exposures were compared in two tracking campaigns, reflects on GPS device choice, privacy, and recruitment strategy.

**Methods:**

Tracking campaigns were conducted in Switzerland and the Netherlands. Participants completed a baseline questionnaire, carried a GPS device (SODAQ) for 2 weeks, and used a smartphone app for a time activity diary. The app also tracked GPS, albeit less frequently. Tracks were combined with air pollution surfaces to quantify NO_2_ and PM_2.5_ exposure by activity.

**Results:**

In Switzerland, participants were recruited from the COVCO-Basel cohort (33% recruitment rate; 489 of 1,475). In the Netherlands, -random recruitment was unsuccessful (1.4% rate; 41 of 3,000). Targeted recruitment with leaflets and a financial incentive (25 Euro voucher) increased participation to 189. Comparisons between smartphone app and SODAQ device data showed moderate to high correlations (R2 > 0.57) for total NO_2_ exposure and NO_2_ exposure at home in both study areas. Activity-specific correlations ranged from 0.43 to 0.63. PM_2.5_ correlations in Switzerland were moderate to high, but lower in the Netherlands (R^2^ = 0.28–0.58), due to smaller spatial contrast in observed PM_2.5_ levels (RMSE < 0.68 µg/m^3^).

**Conclusions:**

Tracking can be effectively conducted using a mobile app or GPS device. The app’s low-frequency GPS readings (every 3–4 min) were sufficient for long-term air pollution exposure assessment. For finer-scale readings, a dedicated GPS device is recommended. Tracking campaigns are crucial for studying personal exposure to air pollution but face challenges due to low recruitment rates and strict privacy regulations. Leveraging an existing cohort can improve recruitment, while targeted leaflet distribution with financial incentives can enhance participation in studies without a pre-recruited group.

**Supplementary Information:**

The online version contains supplementary material available at 10.1186/s12942-025-00405-x.

## Background

Studies investigating the association between long-term health effects and air pollution can typically only consider the residential location of the population [1]. Activity patterns in space and time, which can influence the total exposure a person encounters, are thus ignored. This in turn could lead to exposure misclassification and eventually to bias or loss of precision in epidemiological studies.

Tracking people through space and time can be used to directly assess personal exposure to air pollution. However, a recent review by Hoek et al. [1] only found a handful of exposure studies using GPS tracking to compare residential and time activity integrated air pollution exposures, with study populations ranging from 49 to 393 [2–5].

Different methods are available to obtain location or mobility data from participants in health studies. Chung et al. [6] reviewed GPS tracking methods used in mobility studies aimed at older adults listing three main technologies: GPS-tracking smartphone (apps), smartwatches, and external GPS devices/loggers. In air pollution studies these methods are often combined with a time activity questionnaire to obtain detailed information about temporal activity patterns of the participant which influences the personal exposure. This can be either integrated in the GPS-tracking smartphone app or separately added as a questionnaire [4, 7]. Each method comes with its (dis)advantages. Apps, which require active download and installation by the user on their personal smartphones, can be rolled out relatively easily over a large population. The cost of a smartwatch or external GPS device, however, restricts its application to smaller numbers. Smartwatches and external GPS devices also require more effort for both the researchers and the participants. Researchers need to distribute the devices and maintain them, whereas participants need to remember to switch on and carry the device during the monitoring period and return the device.

Studies using these technologies flagged several issues or limitations such as differences in accuracy and battery drainage, especially when using GPS-enabled smartphones [8]. Other issues that tracking studies struggle with are the generally low recruitment rate, representativeness of the study population and the increasingly stringent privacy laws [9].

In this paper we describe our experience in conducting two tracking campaigns as part of the ‘Accounting for MOBility in AIR pollution exposure estimates in studies on long-term health effects’ (MOBI-AIR) project. MOBI-AIR aimed to investigate whether more sophisticated estimates of individual exposure, considering population mobility, decrease the bias in health studies. The aim of this paper was to give insights on the design of the two tracking campaigns, one each in Switzerland and the Netherlands, highlight the challenges we encountered in the recruitment process, how we addressed privacy law requirements and give recommendations on the choice of GPS tracking methods.

## Methods and data

### Tracking campaign

The initial goal was to recruit 1,000 participants each in Switzerland and the Netherlands and collect detailed information of each subject’s mobility patterns over a 2-week period using a purposely designed mobile phone app and a GPS tracker. No health-related data were collected from the subjects. For this reason, the Dutch part of the tracking campaign received an ethics exemption from the Medische Ethische Toetsingscommissie (METC) Utrecht. The Swiss part of the study was approved by the Ethikkommission Nordwest- und Zentralschweiz (EKNZ) as it recruited from the EKNZ-approved ongoing COVCO-Basel study.

COVCO-Basel is a digital cohort in North-Western Switzerland, specifically initiated to investigate the long-term impact of the COVID-19 pandemic and its containment measures on broad domains of health and wellbeing [10]. Individuals proficient in spoken or written German, aged 18 years or older residing for at least 5 years In Basel-Stadt or Basel-Landschaft, Switzerland, were eligible to participate in the COVCO-Basel study. 12,724 participated at baseline and entered the study between July 2020 and April 2021. The participants who expressed interest to participate in an environmental health study were invited to the MOBI-AIR tracking campaign. In the Netherlands, we did not have an existing cohort from which to recruit thus we started a new cohort.

The tracking campaigns in the two countries were carried out in very similar fashions, via the steps shown in Table 1. In short, after recruitment, potential participants were assessed on their eligibility. If eligible, they were asked to sign an informed consent form. One criterion was the participant’s ability to understand the local official language in the respective study area (German in the Basel region of Switzerland, Dutch in the Netherlands). After signing the informed consent, they were enrolled in the study and asked when they would be available for the 2-week tracking campaign. When confirmed, they were asked to fill in a baseline questionnaire with some general questions about age, sex, income and mobility. After completion of the baseline questionnaire, the GPS device (SODAQ) was sent to the participant together with instructions, including on how to download and operate the mobile phone app. After 2 weeks, the participants were asked to send back the device.

Conforming to the data protection regulations was a major challenge, resulting in substantial delays and personnel efforts in both campaigns. Several steps were included to achieve a privacy by design tracking campaign. Data collection was guided by the principles of data minimisation, pseudonymisation and security. Data minimisation ensured that only necessary personal data were collected via secure online questionnaires, with the app collecting strictly location data and time activity data. All collected data went through meticulous pseudonymisation routines, where each source of data was stored in separate databases with unique key codes: personal information under Personal ID (PID), informed consent under Consent ID (CID), baseline data under app ID (AID), and app data also under AID. Temporary data storage was managed on secure external servers (Qualtrics and Games for Health), with regular downloads to local servers, followed by deletion from the external servers. Permanent storage was maintained in a privacy folder on a high-security local server, with very limited access granted to a select number of investigators. Research data access was restricted to study researchers who could not identify participants. Data transfer was encrypted, and backups were regularly saved and tested. Certified tools (REDCap in Switzerland, Qualtrics in the Netherlands) were used for data collection and processing. Researchers are responsible for data deletion when no longer needed and maintaining data security. Results were published in an aggregated form to prevent identification of individuals. A Data Protection Impact Assessment (DPIA) was conducted, reviewed by privacy officers, and a processor’s agreement was signed with Games for Health, the software company designing the app collecting GPS and TAD data.

**Table 1:** Steps in the tracking campaign in Switzerland and the Netherlands

Steps		Switzerland	The Netherlands
1	Recruitment	Adult participants from the COVCO-Basel cohort, who expressed interest to participate in an environmental health study were sent an invitation email via REDCap (see S3.1 A)	Participants were selected from the general adult population, using physical letters to a random sample of the Dutch population, social media calls and home-delivered letters to selected towns in the province of Utrecht (see S3.1B)
2	Eligibility assessment	On agreeing, potential participants enter a website with unique code and answer several questions to see if they are eligible. Exclusion criteria were as follows: • not capable of answering questions/handling devices; • not strong enough to carry the sensor; • not understanding the local language in Switzerland (German) and in the Netherlands (Dutch); • not in possession of a smartphone (Android 5.0 or higher; iOS 11.0 or higher) and/or internet access; and • not capable of handling smartphone applications.	
3	Informed Consent Form (ICF)	When eligible, the potential participant received an Informed Consent Form by surface mail that they have to sign and send back (see S3.2 A).	When eligible, the potential participant received an Informed Consent Form (ICF) by email that they have to sign via Qualtrics, an online certified survey tool (see S3.2B).
4	Registration and Availability	When the ICF was returned the participant was registered in the system and an email was sent with question about available dates to participate.	When the ICF was signed the participant was registered in the system
5	Baseline Questionnaire	When availability was confirmed the link to the online Baseline- Questionnaire (BLQ) implemented in REDCap was sent by email (see S3.3 A for BLQ)	A BLQ was sent immediately after registration to be filled in via Qualtrics. When the BLQ was finished, an email was sent with the availability for tracking question in groups of registered subjects (see S3.3B for BLQ)
6	Sending Device	Participants with completed BLQ received at the convened measurement period start date the device with instructions for device and app (time-activity questionnaire TAQ) activation by post, plus an email was sent (see S3.4 A)	The device plus a letter with instructions for device and app (time-activity questionnaire TAQ) activation were sent by post, along with an email (see S3.4B)
7	Returning device	After 2 weeks, an email was sent reminding to complete the TAQ and stop the the device.	
8	End of campaign	After receiving the returned device a thank-you email was sent	After receiving the returned device a thank-you email was sent with a voucher of €25.
9	Feedback	After completing the 2-week tracking, each participant received a feedback letter with information about their total and activity–based NO_2_ and PM_2.5_PM_2.5_ exposures, including general advice on how they can reduce their personal air pollution exposure (see S3.5)	

### Tracking devices, GPS measuring

We were initially planning to use the ExpoApp, developed by Donaire-Gonzalez et al. [7], which was specifically designed to track people and collect time activity data. However, since the development of the ExpoApp, and during the pilot phase of this project, a change in the iOS and Android architecture meant that the GPS location functionality drained the phone battery too quickly, such that using a phone app was not an attractive option. In collaboration with the “EXposome Powered tools for healthy living in urbAN Settings” (EXPANSE) project [11], we therefore searched for other solutions.

Firstly, we used a tracker, developed by the Dutch company SODAQ (www.sodaq.com). The tracker was adapted specifically for this project by increasing the position sampling frequency. The GPS tracker recorded 20 s the following: (1) GPS/GLONASS - SGGP.12.4.A.02 (5–20 m); (2) WiFi - ESP8285 (10–50 m); and (3) Cell - NN02-220 (100 m+). The size and weight of the instrument were ~ 80.00 × 80.00 × 20.00 mm and ~ 100 g. The tracking mode was only activated when the onboard accelerometer detected movement. When not in active use (that is, in case of ‘no movement detected’), the tracker went into sleep mode to save power whilst still recording a location every 5 min. The trackers logged all collected location data via an onboard SD card, which was accessible through the USB connector. The trackers sent data ‘near real time’ to the internet, over NB-IoT or LTE-M, directly to secured local servers at Swiss TPH, Basel and IRAS, Utrecht. Trackers connected to LTE-M and NB-loT networks, the 2 main networks in Europe. Trackers were recharged and tested before handing out to each participant.

Secondly, as a bonus, iOS and Android architecture changed, solving the battery drain issue related to GPS data capture, allowing us to take advantage of measuring GPS on the app developed by Games for Health (GfH; https://gamesforhealth.net/), initially only meant to provide a daily time activity questionnaire. The app was able to measure the location using a pin every 3–4 min.

GPS data from both devices were retrieved and cleaned in R, version 4.3.2, relying on the sf package, version 1.0–15 (10.32614/CRAN.package.sf), to manipulate and analyze spatial data. For the SODAQ devices priority was given to GPS data, WiFi was only used when no GPS signal was available. For both devices data points were excluded if the speed between points exceeded 45 m/s or if there were less than 6 data points per hour while on the move. Speeds exceeding 45 m/s (or 162 km/hr) were assumed to be unrealistic as this is the speed limit of trains in Switzerland. We also excluded hours with less than 6 data points as 10 min was our time resolution and with less than 6 points, we would have missing 10 min intervals.

### Exposure estimates

The air pollution exposure modelling for MOBI-AIR is described in detail by de Hoogh et al. [12]. In brief, two different approaches were used for the exposure modeling in each country. In Switzerland, existing spatiotemporal geostatistical models [13, 14] estimating annual average PM_2.5_ and NO_2_ surfaces, at a fine spatial resolution of 100 × 100 m for the year 2016, were rescaled to long-term hourly weekday and weekend day surfaces. For rescaling, we followed a method previously applied by de Nazelle et al. [15]. Measurements at 9 PM_2.5_ and 63 NO_2_ background monitoring stations across Switzerland were used to calculate diurnal ratios by dividing weekend and weekday averaged hours (1 to 24 h) with annually averaged hours. These ratios were then applied to the 100 × 100 m PM_2.5_ and NO_2_ concentration surfaces resulting in long-term hourly weekday and weekend day surfaces. In the Netherlands, Ndiaye et al. [16] developed maps of annual average hourly concentrations of PM_2.5_ and NO_2_ using supervised linear regression (SLR) and random forest (RF) models based upon air quality monitoring data (*n* = 544 for NO_2_; *n* = 227 for PM_2.5_) in the Netherlands and the neighbouring countries Belgium and Germany. Long-term hourly weekday and weekend day NO_2_ and PM_2.5_ concentrations were modelled for the years 2016–2019 combined, for two seasons (cold and warm) using predictor variables including land use, roads, meteorology, population and satellite retrievals and chemical transport model estimates. SLR and RF models performed similarly, with 5-fold cross validation R^2^ for the hourly models between 0.50 and 0.78 and 0.24–0.62 for NO_2_ and PM_2.5_ respectively. In this study we used the hourly surfaces derived from the RF models.

### Exposure assessment

The GPS locations for individual participants of the tracking campaigns measured by the mobile phone app and the SODAQ device were overlaid with the modelled hourly NO_2_ and PM_2.5_ surfaces. As data points were not collected in the same time intervals, each data point was assigned a duration, calculated as half the time difference to the previous point plus half the time difference to the next point. The duration was then used to calculate time weighted hourly exposure means.

### Data management and analysis

Data management in Switzerland was conducted in REDCap used for cohort management and data collection in the ongoing COVCO-Basel cohort, whereas in the Netherlands MS Access and Qualtrics was used. Data from each SODAQ device were linked to the corresponding user by matching International Mobile Equipment Identity (IMEI) numbers with start and end dates. IMEI numbers were recorded in REDCap (CH) and in MS Access (NL) when devices were distributed, and participants were asked to enter usage start and end times in questionnaires. All individual files were consolidated into a comprehensive database. For app data, we first parsed the JSON (JavaScript Object Notation) files and applied the same linkage method as for SODAQ devices, matching app IDs (AID) to participant IDs (OIDs) and subsetting the data to the relevant time periods. Subsequently, all time activity data collected through the app, saved as separate JSON files, were downloaded, parsed, and linked to the OIDs. This dataset was then merged with the app and SODAQ files, ensuring each measurement was associated with the correct time activity via a non-equijoin using OID and time period. Next, we combined the app and SODAQ data, including an indicator variable to retain source information. Records with missing time activity data were excluded from the analysis. SODAQ devices exhibited unreliable performance indoors (e.g., no GPS fix, forgot to recharge battery) similar to app data issues (e.g., phones in flight mode or switched off). To address this, we used time activity data and home address information from baseline questionnaires to impute missing data for periods when participants were at home. One location point per hour was imputed for each missing hour, as hourly exposure surfaces were utilized. This integrated dataset was then overlaid with hourly exposure surfaces (differentiated by weekdays and weekends) using the sf and terra packages in R to extract exposures for each point and assign final exposure values based on matching hours and day of week. Time-weighted outdoor exposures were calculated for each activity, as well as total outdoor exposures by aggregating across all activities (home/other, socializing, travel and works/school extracted from the time-activity diaries) for each participant.

## Results

### Recruitment and communication procedures

A total of 489 people participated in the Basel tracking campaign during 2 waves (Sep-Dec 2022 and Jan-May 2023). 1,475 people of the COVCO-Basel study were invited by email, 502 signed the informed consent form, and finally 489 participated (33% recruitment rate). The relatively high recruitment rate builds on the vast cohort experience available in COVCO-Basel and the active and labor-intensive communication with COVCO-Basel participants by phone and email. However, if we include the recruitment of the COVCO-Basel cohort (1,475 participants of 112,724 invited people) we could also recalculate this to a more realistic and much lower recruitment rate of 489 / 112,484 = 0.4%.

**Fig. 1:**
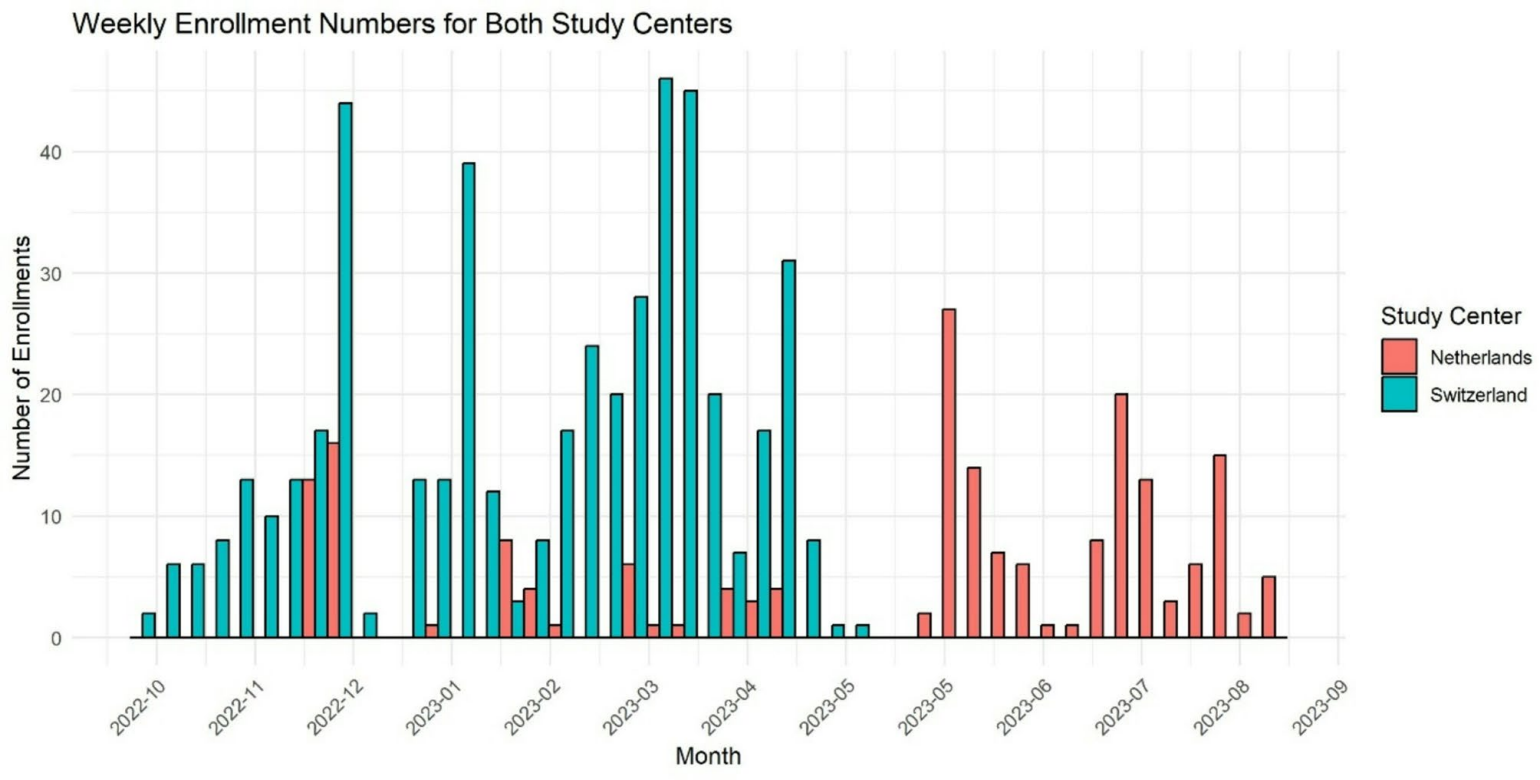
The number of participants enrolled in the study each week for each study center The x-axis represents the weeks of the year, while the y-axis shows the number of enrollments during each week (blue bars for Switzerland; red bars for the Netherlands)

In the Netherlands, we started with a random population survey whereby 3000 letter invitations were sent in September 2022 of which 41 responded (1.4% response rate). We used the Dutch address database (BAG) in an attempt to select a random population. The database does not contain names nor personal characteristics, so the letter was addressed to “the occupant”. This low response prompted new recruitment strategies through social media and a distribution of 500 leaflets in the city of Utrecht (capturing 73 more) and expanding inclusivity to individuals who do not speak the local language but have a functional understanding of English, leading to translating all materials to English. Based on advice from social scientists in Utrecht University working with surveys, we included a 25 Euro voucher as an incentive to improve response. We subsequently delivered 5000 more leaflets in the city and province of Utrecht in May 2023 in selected low and high socio-economic status neighborhoods in the city and selected surrounding towns. This resulted in a total of 189 participants enrolling in the tracking campaign which took place in 7 waves between November 2022 and August 2023, thus covering winter and summer seasons. Most subjects were measured between May and August 2023. Figure 1 shows the enrollment of participants in Switzerland and the Netherlands.

Both campaigns implemented personalized feedback so that each participant received an email summarizing their personal exposure after the completion of the tracking campaign. (See Supplement A for the English version of the template letter used in Switzerland).

To assess the representativeness of these campaigns, national demographic data for the Swiss and Dutch populations were included for comparison (Table 2).

**Table 2:** Demographics of participants in the Swiss (CH) and Dutch (NL) tracking campaigns compared to National population data (percentages in each category calculated for total population)

		Switzerland				The Netherlands			
		Track	%	National^1^	%	Track	%	National^2^	%
N		489		8,815,385		189		17,590,672	
Sex	Male	195	40	4,379,953	50	84	44	8,745,468	50
	Female	293	60	4,435,432	50	103	54	8,845,204	50
	Other	1	0			2	1		
Age	18–40	90	18	2,465,145	28	55	29	4,487,841	36
	40–60	268	55	2,513,375	29	71	38	3,415,571	27
	> 60	131	27	2,253,637	26	63	33	4,683,950	37
Yearly	< 36’000 CHF / <25’000€	22	4	1,615,440	18^3^	18	10	6,134,100	35
Gross	36’-72’000/ 25’-50’000€	86	18	1,681,480	19^3^	44	23	4,959,000	28
Income	> 72’000 CHF / >50’000€	348	71	1,732,280	20^3^	100	53	2,273,300	13
	No answer	33	7			27	14		
Education	Pre high school	125	26	706,950	15	18	10	1,959,000	20
	High school	34	7	2,167,980	46	14	7	3,720,000	38
	Higher Education	336	69	1,838,070	39	157	83	4,007,000	41
Employment	Fulltime	291	60	2,961,000	34	97	51	5,064,000	29
	Part time / Irregular	116	24	1,752,000	20	37	20	4,673,000	27
	Homemaker / Not Working	27	6			16	8		
	Retired	44	9	2,115,692	24	36	19	3,578,513^4^	20
	Other / No Answer	11	2			4	2		

^1^www.bfs.admin.ch; data for 2022

^2^www.cbs.nl; data for 2022 (expect for income– data from 2020)

^3^https://de.statista.com/statistik/daten/studie/291841/umfrage/einkommensverteilung-in-der-schweiz/ Verteilung der Beschäftigten in der Schweiz nach Netto-Lohnhöhenklassen im Jahr 2020, Bundesamt für Statistik

^4^www.svb.nl; data for 2022

Females were overrepresented in both campaigns. Most participants were employed, with a smaller proportion being retired. Individuals with higher education levels and higher incomes were also overrepresented. When compared to national demographic data, it was clear that the tracking campaigns did not fully represent either population. Participants aged 40–60, those with higher incomes, higher education levels, and full-time employment were overrepresented in both countries. However, the tracking campaigns did include a reasonable representation of other age groups, as well as participants in low to medium education and income brackets, and those who were not employed.

### Time activity data

Based on the time activity data, participants in both campaigns spend most of their time at home (~ 60%). Time spent at work or school was 16% in Switzerland compared to 10% in the Netherlands. Time spent in travel and socializing activities were again comparable in both campaigns with app. 8% and 9% respectively (Table 3).

**Table 3:** Percentage of time per activity obtained from the tracking campaigns in Switzerland and the Netherlands

Activity	Switzerland	The Netherlands
Home / Other	59.9	62.7
Work / School	16.2	10.1
Travel	8.1	7.5
Socializing	10.2	8.2
Missing	5.6	11.5

In the Dutch tracking campaign, the share of transport modes, collected in the time activity data (Table 4), matched the national figures quite well. This, however, was not the case for Switzerland. In Switzerland, participants of the tracking campaign, travelled half as much by car and walking (21% v 41% nationally) and more by public transport (32% v 11%) and by bicycle (26% v 7%) (Table 4).

**Table 4:** Comparison of percentage of time per commuter mode obtained from the tracking campaigns with National figures for Switzerland and the Netherlands

Mode	Switzerland		The Netherlands	
	Tracking campaign	National^1^	Tracking campaign	National^2^
Walking	20.9	41.1	17.8	23.5
Bike	26.3	6.8	38.3	24.1
Public transport	31.5	11.0	9.1	9.7
Car	21.3	41.1	34.8	42.7

1) https://www.mobilitaetsverhalten.bfs.admin.ch/en/, 2021

2) https://www.cbs.nl/nl-nl/visualisaties/verkeer-en-vervoer/personen/hoeveel-reisden-inwoners-van-nederland-en-hoe

Compared to Switzerland as a whole, the Basel study area has a more urban character as it includes the cantons Basel-Stadt (mostly urban) and Basel-Landschaft (mix urban-rural) with a well-connected and extensive public transport system, likely explaining this mismatch. In the Dutch tracking campaign, participants used the car modestly less and cycled modestly more than the national average. This is possibly related to differences between the Utrecht province study area, where the majority of the participants resided, and the entire country and the higher education / income of the tracking population.

### GPS data

In both study areas all participants were asked to use both the mobile phone app and the SODAQ device simultaneously to track their location. As explained in Sect. 2.2, the two tracking methods had different data capture intervals and behaviour.

We imputed missing GPS readings during the time when participants were at home, resulting in a small number of tracking points that needed to be imputed (in Switzerland 2% of the total tracking points were imputed, in the Netherlands 1%).

### Comparison exposures based on GPS readings from app and SODAQ device

**Fig. 2:**
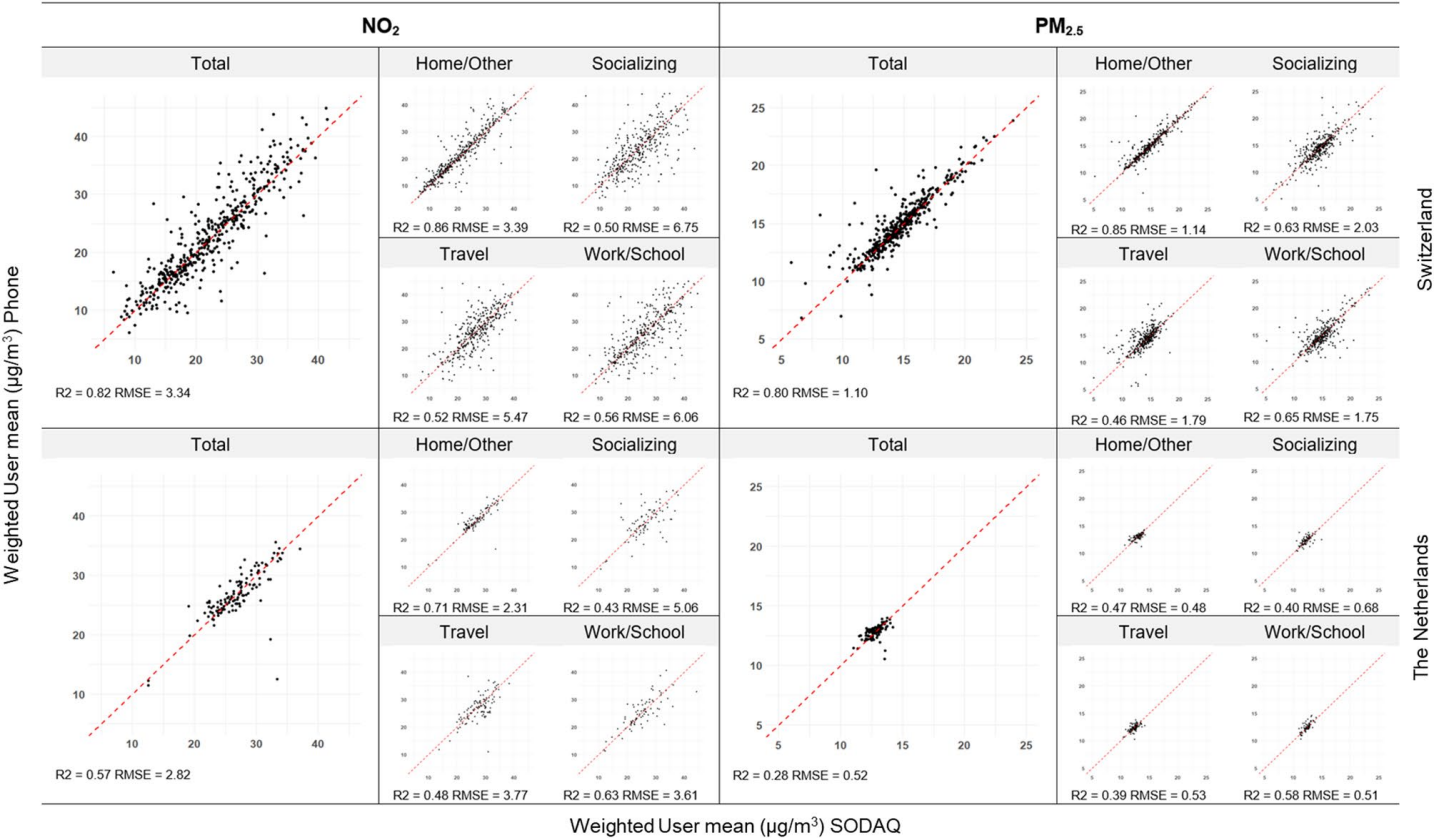
Participation-level comparison of NO_2_ and PM_2.5_ exposures based on GPS readings from the mobile phone app and the SODAQ device, by activity NO_2_ and PM_2.5_ exposures were calculated and compared over the whole period (total) and by activity (Home, Socializing, Work/School and Travel). Each point in the scatterplots is a participant of the Swiss (top row) and the Dutch (bottom row) tracking campaign

**Fig. 3:**
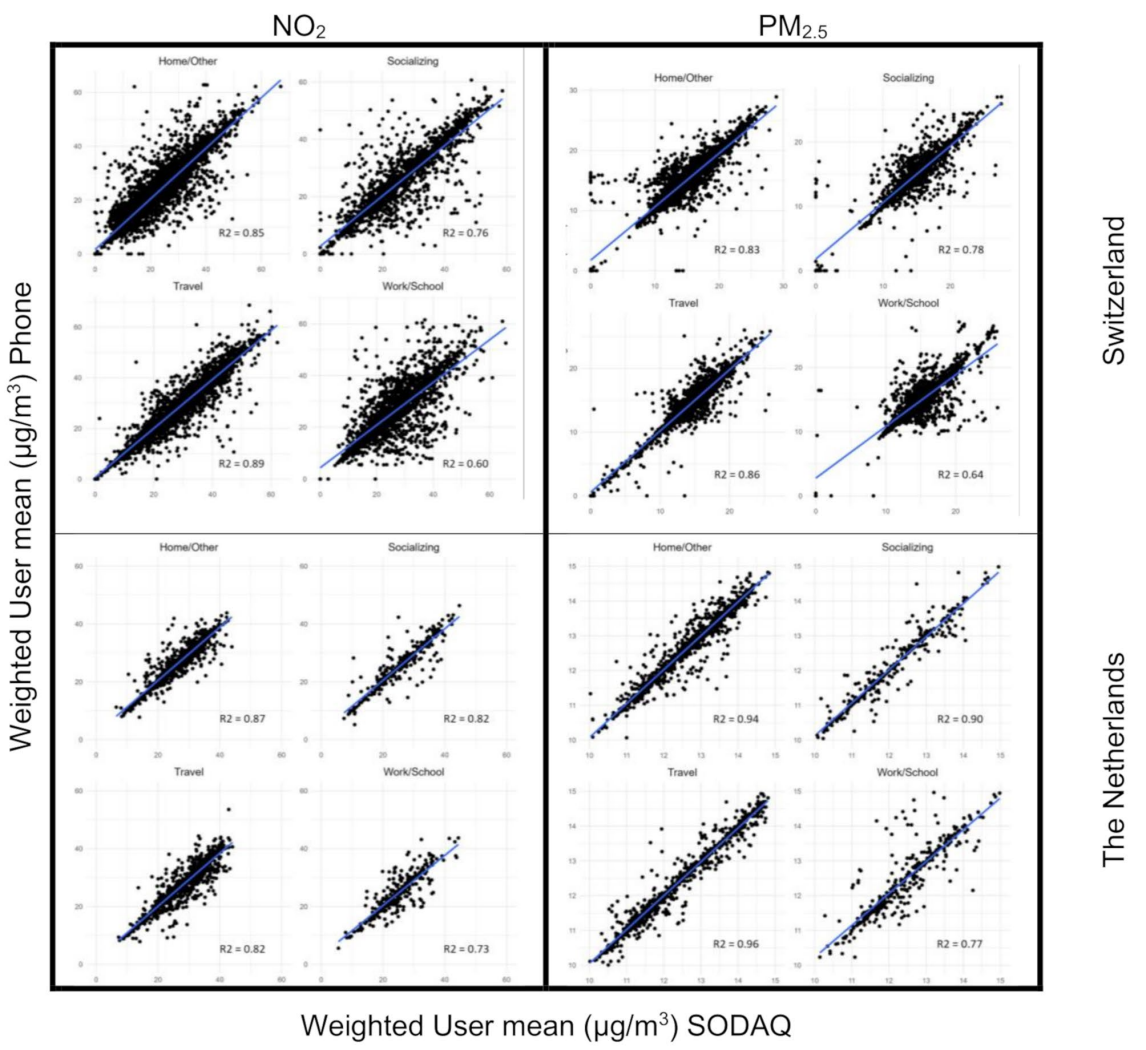
Activity-level comparison of NO_2_ and PM_2.5_ exposures based on GPS readings from the mobile phone app and the SODAQ device NO_2_ and PM_2.5_ exposures were calculated and compared over the whole period (total) and by activity (Home, Socializing, Work/School and Travel). Each point in the scatterplots is an activity of the Swiss (to row) and the Dutch (bottom row) tracking campaign

We compared the computed time-weighted air pollution exposures aggregated by participant (Fig. 2) and aggregated by activity (Fig. 3) using the app and the SODAQ GPS readings in both tracking campaigns. A good agreement was found between NO_2_ exposures from the 2 different GPS capturing methods by participant (Fig. 2) for Switzerland (R^2^ = 0.82) for all activities together (Total) but less so for the Netherlands (R^2^ = 0.57). The RMSE was lower in the Netherlands (2.83 µg/m^3^) compared to Switzerland (3.34 µg/m^3^), indicating less variability in the Netherlands. Similar patterns between the two countries, with less variability in the Netherlands than in Switzerland, were found when split by activity. Specifically, we found moderate correlations for Socializing, Travel and Work/School (R^2^ = 0.43–0.63), and high correlations for Home/Other (R^2^ = 0.86 in CH and R^2^ = 0.71 in NL). The agreement between the 2 GPS capturing methods was smaller for PM_2.5_, especially in the Netherlands (Total: R^2^ = 0.80 in CH; R^2^ = 0.28 in NL). Correlations by activity followed a similar pattern as with NO_2_, with higher correlations for Travel than the other three activities. RMSE for PM_2.5_ in both countries were small (< 2.03 µg/m^3^), but again smaller in the Netherlands (< 0.68 µg/m^3^). A good agreement was found between NO_2_ and PM_2.5_ exposures from the 2 different GPS capture methods when aggregating by activity with R^2^s between 0.60 and 0.96 (Fig. 3). For a small number of individual participants large differences were found.

In a few cases either the SODAQ device or the mobile phone app recorded only very few GPS data points. In Fig. 2 we can see these outliers in the graph of the total exposure. The outliers represent participants who had incomplete measurement data from one device. This can result in a very different total exposure, as one dataset represents a smaller time window, while the other dataset covers more points (and therefore more time and space). The plots by different activities for both countries show higher correlations between the 2 GPS measurements for Home/Other compared to the other activities, the only exception being Work/School PM_2.5_ exposure in the Netherlands which had a higher R^2^. Outlier points that have lots of missing data thus may not be represented in all activities. When looking into single activity blocks (Fig. 2), this is even more obvious, because only activity blocks with data points from both devices are represented.

## Discussion

Recruitment of participants posed a major challenge in our study. In Switzerland we were able to recruit from COVCO-Basel, an existing cohort, individuals who had indicated that they were willing to participate in further environmental research. This led to a relatively high recruitment rate of 33% and 489 participants in the Swiss tracking campaign. However we have to acknowledge that the initial COVCO-Basel study invited 112,848 people of which 12,724 agreed to take part [17]. Of these 12,724 people, 1,475 were willing to participate further in environmental research, and of those 489 people participated in MOBI-AIR. So instead of the earlier mentioned 33% recruitment rate, we could also recalculate this to a more realistic and much lower recruitment rate of 489 / 112,484 = 0.4%. The COVCO-Basel cohort was biased with woman respectively under- and over-represented in the < 65 and + 65 age groups, and foreigners underrepresented due to language of the questionnaire being in German.

In the Netherlands we were not able to recruit from an existing cohort, starting instead with a random sample of 3,000 people from the Dutch population. Only a disappointing 1.4% of this random sample agreed to take part. Upscaling our efforts using social media also did not lead to significant gains. We therefore used more a targeted method of spreading 5,000 leaflets in the Utrecht region and adding a financial incentive of 25 Euros. Due to GDPR compliances, we subsequently lost people. In total, 380 people showed an interest, of whom 248 signed an informed consent letter and 208 completed the baseline questionnaire. Some participants who already agreed to participate did not complete tracking, some even after receiving the equipment. Ultimately 167 participants successfully completing the Dutch tracking campaign. The lower number compared to the Swiss campaign is likely related to the lack of a recent existing cohort from which to start recruitment. Using an existing cohort that was recently recruited to pull from has a clear advantage for recruitment in environmental studies. Although we did not succeed in recruiting the initial aim of 1,000 participants, both campaigns - with almost 500 (Swiss) and 200 (Dutch) - can be considered large tracking studies compared to previous air pollution tracking studies that included between 41 and 393 participants [2–5, 7, 18–20] None of these previous tracking studies report on the issues they encountered in the recruitment process. Only 2 studies reported recruitment rates (0.6% (5) and 3.4% (4)) which were comparable to our Dutch study (1.4%). In general, participation rates in health surveys have been decreasing over time [21, 22] leading to possible bias in the population sample. Three out eight studies used a smartphone app with GPS tracking capability [4, 5, 7, 19, 20] and the other three a GPS tracking device [2, 3, 18].

The full Swiss and Dutch population were not well represented in both the tracking campaigns, with the 40–60 age, higher education, high income and fulltime workers groups being overrepresented in the tracking campaigns. Unfortunately, we were unable to influence the recruitment process to better align the study population of our tracking campaigns with the general population. The overrepresentation of highly educated participants, however, aligns with patterns observed in most exposure and epidemiological studies that rely on voluntary participation. In our study we choose to use the local official language in the Netherlands (Dutch) and in the Basel region of Switzerland (German). This might have led to missing those people who do not speak these languages (mainly foreigners) and for that reason our language prerequisite could have been a barrier for recruitment.

Participants in the campaigns were tracked using two methods: (1) a wearable tracking device (SODAQ) and (2) a mobile phone app with GPS functionality. As outlined in Sect. 2.2, the initial plan to use an existing mobile app was hindered by significant battery drain caused by GPS functionality on both iOS and Android devices at the start of the project. Consequently, the protocol was adjusted to incorporate the wearable SODAQ device, which offered the added benefit of providing high-frequency GPS readings every 20 s. The mobile app was primarily designed to collect time-activity data from participants. Subsequent advancements in iOS and Android architectures enabled the app to perform GPS measurements, though at a lower temporal frequency of 3–4 min. This allowed both the SODAQ device and the app to be used in the study, enabling comparisons between the two methods. The study found moderate to strong correlations between total NO₂ exposures measured by the app and the SODAQ device across the two study areas. However, correlations were lower when examined by activity type (e.g., Socializing, Work/School, Travel), except for when participants were at home. For PM_2.5_, the correlations between the two methods were generally weaker in both countries and across all activities. This is primarily due to smaller spatial variability in PM_2.5_ concentrations in both countries, as shown by the low RMSE’s (Fig. 2). Another reason relates to the differences in GPS point frequency, especially noticeable during activities like travel. Figure 1 illustrates a typical bicycle commute, highlighting the disparity in GPS point density when overlaid on the 100 × 100 m air pollution surface, potentially leading to differences in calculated mean hourly exposure whilst travelling. We found that the mobile phone app with GPS functionality deployed in our study is scalable to large cohorts without relying on costly GPS tracking devices.

One potential limitation is that the tracking campaigns were initiated shortly after COVID-19 pandemic lockdowns and restrictions were eased. To address this concern, a thorough evaluation of travel trends using data from Google and TomTom was performed in both study areas. This analysis confirmed that travel activity had stabilized and returned to levels comparable to those observed before the pandemic.

## Conclusions

Tracking campaigns are valuable for air pollution health studies to gain insight in personal exposure. However, low recruitment rates and increasingly stringent privacy or GDPR regulations make them more and more difficult to conduct. Using an existing, recently recruited cohort (COVCO-Basel in Switzerland) was successful in bolstering recruitment rates. When no existing cohort is available to recruit from, like in the Netherlands, a targeted approach using leaflets in selected areas with a financial reward can help bolster the recruitment rate.

Tracking campaigns can be successfully conducted using either a mobile phone app or a wearable GPS device. The mobile phone app with low frequency of GPS readings (every 3–4 min) turned out to be sufficient for our purpose. For studies requiring finer scale readings, a purposely built GPS device is recommended. For large populations, the app is the more scalable solution.

## Electronic supplementary material

Below is the link to the electronic supplementary material.

Supplementary Material 1

## Data Availability

No datasets were generated or analysed during the current study.
