# Supplementary material for: GPS tracking methods for spatiotemporal air pollution exposure assessment: comparison and challenges in study implementation
Source: Int J Health Geogr. 2025 Jul 26;24:17. doi: 10.1186/s12942-025-00405-x (PMC12296587; doi:10.1186/s12942-025-00405-x)

Supplement A: Example feedback letter (translated from German to English) for the Swiss tracking campaign. Text marked in yellow is personal information


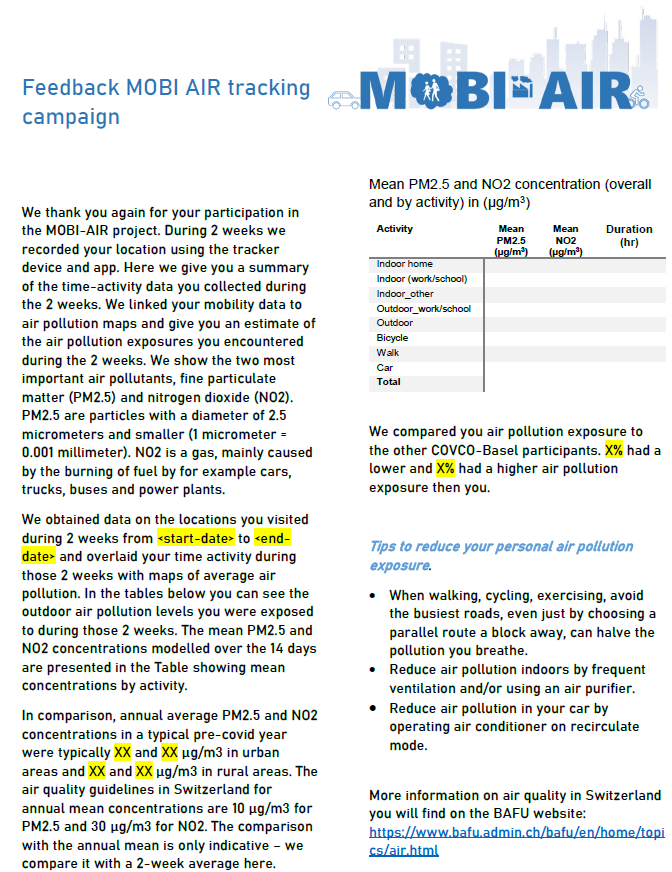

Supplement: Supplementary file 1 — Supplementary Material 1 [file 12942_2025_405_MOESM1_ESM.docx]
